# Dissection of the *qTGW1.1* region into two tightly-linked minor QTLs having stable effects for grain weight in rice

**DOI:** 10.1186/s12863-016-0410-5

**Published:** 2016-06-30

**Authors:** Hong-Wei Zhang, Ye-Yang Fan, Yu-Jun Zhu, Jun-Yu Chen, Si-Bin Yu, Jie-Yun Zhuang

**Affiliations:** State Key Laboratory of Rice Biology and Chinese National Center for Rice Improvement, China National Rice Research Institute, Hangzhou, 310006 China; State Key Laboratory of Crop Genetic Improvement and National Center of Plant Gene Research (Wuhan), Huazhong Agricultural University, Wuhan, 430070 China

**Keywords:** Close linkage, Genotype-by-environment interaction, Grain size, Grain weight, Minor effect, Quantitative trait locus, Rice

## Abstract

**Background:**

Most agronomical traits of crop species are complex traits controlled by multiple genes and affected by environmental factors. While considerable efforts have been made to fine-map and clone major quantitative trait loci (QTLs) for yield-related traits in rice, it is not until recently that the attention has been paid to minor QTLs. Following previous dissection of QTLs for grain weight and grain size in a 12-Mb interval on the long arm of chromosome 1 in rice, this study targeted at one putative QTL region for a more precise mapping and for analyzing the genotype-by-environment interaction of minor QTLs.

**Results:**

Four BC_2_F_10_ plants of the *indica* rice cross ZS97///ZS97//ZS97/MY46 were selected. They carried overlapped heterozygous segments that jointly covered the entire putative region for *qTGW1.1* detected previously. Four sets of near isogenic lines (NILs) were developed from selfing progenies of the four plants. Each NIL set consisted of 32 ZS97 homozygous lines and 32 MY46 homozygous lines that differed in the corresponding heterozygous region. They were grown in two locations having distinct ecological conditions and measured for 1000-grain weight, grain length and grain width. Two QTLs were separated in an 835.2-kb interval flanked by DNA markers Wn28447 and RM11569. They both showed consistent effects across the two environments. The *qTGW1.1a* located within the 120.4-kb interval Wn28447 − RM11543 significantly affect all the three traits with the enhancing allele derived from ZS97, showing a stronger influence on grain weight than on grain length and width. The *qTGW1.1b* located in the 521.8-kb interval RM11554 − RM11569 significantly affect grain weight and length with the enhancing allele derived from MY46, having a stronger influence on grain length than on grain weight. Consistent performance of the two QTLs was confirmed in a validation experiment using five NIL-F_2_ populations segregated for either *qTGW1.1a* or *qTGW1.1b*.

**Conclusion:**

Separation of closely-linked QTLs having small effects is achievable in the absence of major-QTL segregation. Minor QTLs for complex traits could act consistently in diverse environments, offering the potential of pyramiding beneficial alleles of multiple minor QTLs through marker-assisted selection.

**Electronic supplementary material:**

The online version of this article (doi:10.1186/s12863-016-0410-5) contains supplementary material, which is available to authorized users.

## Background

Most agronomical traits of crop species are complex traits controlled by multiple genes and heavily influenced by environmental factors. Since the past decade, characterization of quantitative trait loci (QTLs) underlying complex traits in rice has been moved from primary mapping to fine-mapping and cloning. A considerable number of QTLs having large effects on heading date, grain number or grain weight were cloned [1–3]. More recently, the attention has also been paid to QTLs that have relatively small effects. For heading date which has been taking the leading position in rice QTL cloning, two minor QTLs were cloned [4, 5] and three more were fine-mapped [6–8]. It was shown that a minor QTL could have a consistent performance across different genetic backgrounds and environmental conditions [6].

The performance of grain yield and its component traits in rice often varies greatly with the change of environmental conditions, thus QTLs controlling these traits are generally recognized to be highly sensitive to environments. Nevertheless, such an instable performance may in fact result from the interactions between environmental factors and QTLs for other traits such as heading date [9]. Indeed, major QTLs controlling heading date and photoperiod sensitivity in rice have been commonly found to have pleiotropic effects on grain number and grain yield, thus the performance of yield traits varied greatly under short-day and long-day conditions [10–14]. When this type of QTLs is homozygous in a population so that the genotype-by-environment (GE) interaction for yield traits due to gene pleiotropism is eliminated, it should be possible to identify QTLs for yield traits that have stable effects under different environmental conditions.

Panicle number, grain number and grain weight are the three main determinants of grain yield in rice, of which grain weight is the trait having the most stable performance [15, 16]. Thus, it is reasonable to firstly target on grain weight for identifying QTLs having small effects for yield traits. In our previous studies, detection of minor QTLs for this trait was conducted using segregating populations derived from the *indica* rice cross Zhenshan 97 (ZS97)///ZS97//ZS97/Milyang46 (MY46). Four minor QTLs, designated as *qTGW1.1*, *qTGW1.2a*, *qTGW1.2b*, and *qTGW1.2c*, were resolved in a 12-Mb interval on the long arm of chromosome 1 [17–19]. These QTLs showed no significant effects on heading date and were consistently detected in different environments and populations. Nevertheless, the QTLs regions identified were fairly large and the effects of two or more QTLs were often mixed in the same population. More precise mapping of the QTLs and having them tested individually in different environments would provide a better determination of their performances.

In this study, four sets of near isogenic lines (NILs) with sequential segregating regions covering or neighboring to *qTGW1.1* were tested in two distinct environments. Two QTLs having no significant GE interactions were separated. The *qTGW1.1a* was located within a 120.4-kb region flanked by InDel marker Wn28447 and SSR (simple sequence repeat) marker RM11543, and *qTGW1.1b* was situated within the 521.8-kb interval RM11554 − RM11569. Afterwards, five NIL-F_2_ populations were developed, using which the effects of *qTGW1.1a* and *qTGW1.1b* were confirmed.

## Methods

### Rice materials

**Fig. 1:**
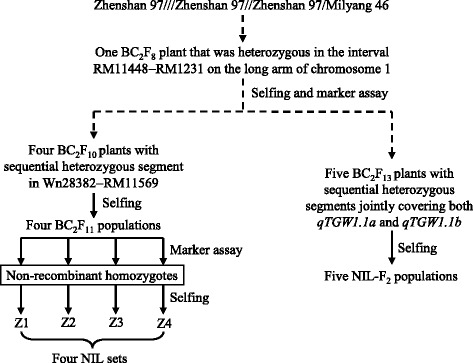
Development of the rice populations used in this study

Four sets of NILs and five NIL-F_2_ populations were used in this study. They were derived from a BC_2_F_8_ plant of the rice cross ZS97^3^/MY46 as described below and summarized in Fig. 1. All the rice materials, including the source populations from BC_2_F_1_ through BC_2_F_8_, were developed at the China National Rice Research Institute.

**Fig. 2:**
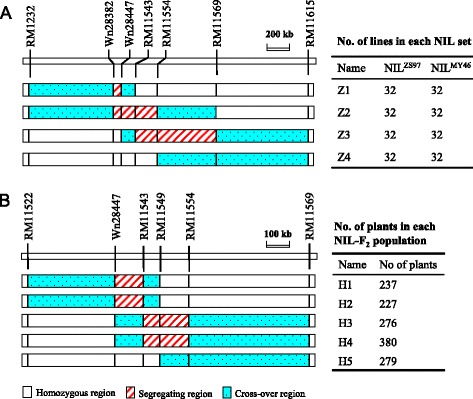
Genotypic compositions of the rice populations in the target region. **a** Four sets of NIL pairs. **b** Five NIL-F_2_ populations

In the original mapping of *qTGW1.1* [17], RM11448 − RM1231 was the region showing segregation for this QTL in the BC_2_F_6_ and BC_2_F_7_ populations of ZS97^3^/MY46. Thus, a BC_2_F_8_ plant carrying a heterozygous segment extending from RM11448 to RM1231 was selected. This plant was selfed and advanced to the BC_2_F_10_ generation, with marker assay performed and pedigree information documented. Following updated information of the *qTGW1.1* location [19], four BC_2_F_10_ plants with sequential heterozygous segments extending from Wn28382 to RM11569 were identified. Altogether, 255 polymorphic SSR markers were tested, including 20 markers located in the RM11448 − RM1231 interval and 235 markers in other regions. The four BC_2_F_10_ plants were homozygous in all regions outside Wn28382 − RM11569. In the resultant BC_2_F_11_ populations, homozygous plants with no recombination in the corresponding segregating regions were selected. Selfing seeds of these plants resulted in the development of four sets of NILs, namely, Z1, Z2, Z3 and Z4, which consisted of 32 ZS97 homozygous lines and 32 MY46 homozygous lines that differed in the regions covering Wn28382 − Wn28447, Wn28382 − RM11554, RM11543 − RM11569 and RM11569, respectively (Fig. 2a).

Since analyses using the four NIL sets indicated that two QTLs for grain weight were located in the target region, other populations were developed for a cross-validation. Five BC_2_F_13_ plants of ZS97^3^/MY46 with sequential heterozygous segments that jointly covered both the *qTGW1.1a* and *qTGW1.1b* regions were selected. They were selfed to produce five NIL-F_2_ populations, namely, H1, H2, H3, H4 and H5, which consisted of 237, 227, 276, 380 and 279 plants segregated in the regions covering Wn28447 − RM11543, Wn28447 − RM11543, RM11543 − RM11554, RM11543 − RM11554 and RM11554, respectively (Fig. 2b).

### Field experiment and trait evaluation

The four NIL sets were grown in Hangzhou, Zhejiang (30° 04′ N, 119° 54′ E) from May to September (ZJ trial) in 2014 and Lingshui, Hainan (18° 31′ N, 110° 00′ E) from December 2014 to April 2015 (HN trial). The day-length conditions in the ZJ and HN trials are long-day and short-day, respectively [20]. The experiments followed a randomized complete block design with two replications. For each replication, twelve plants per line were planted in one row with spacing of 16.7 cm between plants and 26.7 cm between rows. At maturity, five of the middle ten plants in each row were harvested in bulk and sun-dried. The grains were soaked in 3.5 mol/L NaCl solution and floating grains were removed. The fully filled grains were washed with clean water and dried in oven at 37 °C for 16 h. After cooling to the ambient temperature, approximately 600 grains were randomly selected and weighted. Three traits, 1000-grain weight (TGW), grain length (GL) and grain width (GW), were measured using a automatic seed counting and analyzing instrument (Model SC-G, Wanshen Ltd, Hangzhou, China). Mean values over two replications were used for data analysis.

The five NIL-F_2_ populations were planted in Hangzhou from May to September in 2015. The planting density was 16.7 cm between plants and 26.7 cm between rows. At maturity, plants were harvested individually and sun-dried. Approximately 200 fully filled grains of each plant were randomly selected for the measurement of TGW, GL and GW.

### DNA marker analysis

Total DNA was extracted following the method of Zheng et al. [21]. PCR amplification was performed according to Chen et al. [22]. The PCR products was visualized on 2 % agarose gels using Gelred staining for RM11554 and on 6 % non-denaturing polyacrylamide gels using silver staining for other markers. All the SSR markers were selected from the Gramene database (http://www.gramene.org/). The Indel markers Wn28382 (Forward primer: 5′-TGGTGACCCAATTAAACGGTA-3′; Reverse primer: 5′-AAATTTTGGGAACATGAAAGGCTC-3′) and Wn28447 (Forward primer: 5′-AAAGTCACTAGCATATGGG-3′; Reverse primer: 5′-CTCAATTGTGAAACCGGGTA-3′) were designed according to the difference between ZS97 and MY46 detected by whole-genome re-sequencing.

Polymorphic DNA markers located in the target region were used to identify desirable plants for constructing the four NIL sets and five NIL-F_2_ populations. In QTL mapping using the five NIL-F_2_ populations, individual plants of the population H5 were genotyped using the only segregating marker RM11554, and those of other populations were assayed with two flanking markers of the segregating regions, *i.e.*, Wn28447 and RM11543 for H1 and H2, and RM11543 and RM11554 for H3 and H4.

### Data analysis

For the four NIL populations, two-way analysis of variance (ANOVA) was performed using SAS procedure GLM [23] as described previously [24]. In the analysis, a mixed model Genotype + Line (Genotype) + Environment + Environment*Genotype was applied. When a significant difference (*P* < 0.05) was detected between the two genotypes, the same data were used to estimate the genetic effect of the QTL, including additive effect and the proportion of phenotypic variance explained by the QTL (*R*^2^).

For the NIL-F_2_ populations, genetic distance between markers was calculated with Mapmaker/Exp 3.0 [25] and presented in centiMorgan (cM) derived using the Kosambi function. QTL analysis was performed with Windows QTL Cartographer 2.5 [26], using interval mapping for populations H1, H2, H3 and H4, and single-marker analysis for H5.

## Results

### Phenotypic performance of the four NIL populations in two locations

**Fig. 3:**
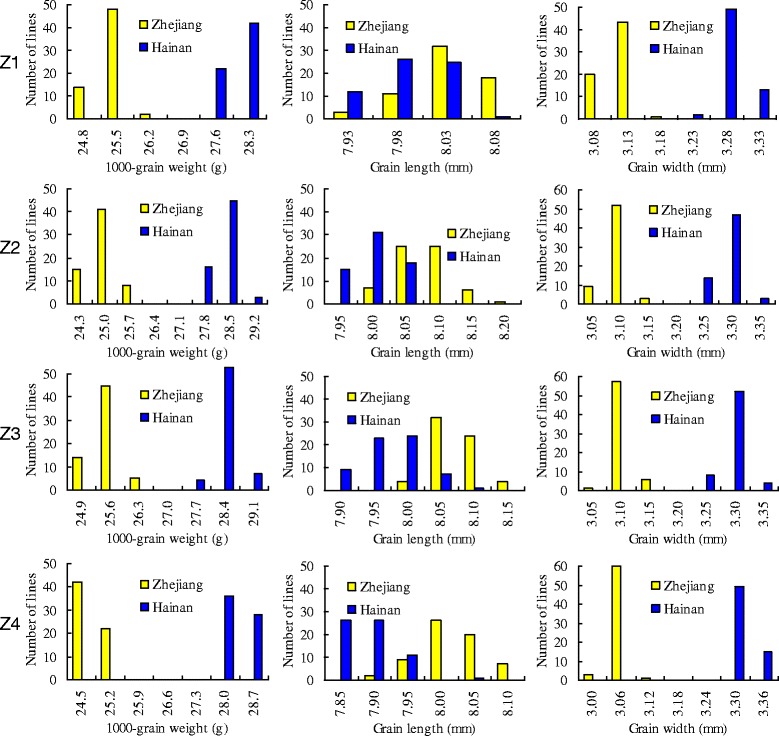
Phenotypic distributions of the four NIL populations in the two trials conducted in Zhejiang and Hainan provinces of China, respectively

In the ZJ and HN trials, the three traits all showed continuous distribution with low variation in the four NIL populations (Fig. 3). Meanwhile, the trait values showed large differences between the two locations, indicating that the environmental difference had a great effect on the traits under study. Two of the traits, TGW and GW, had discrete phenotypic values between the two locations, and the values were all higher in the HN trial than in the ZJ trial. In addition, the difference in each population was much more distinct between the two locations than among the 64 rice lines. These results indicate that genes for grain weight and grain size that were not segregated in these populations had a strong response to the environmental variations between the two locations.

Partitioning of the phenotypic variance of TGW, GL and GW using two-way ANOVA is presented in Additional file 1 and the significance levels of the *F*-test’s *p*-values are shown in Table 1. The variation between the two environments was highly significant (*P* < 0.0001) for all the three traits in all the four populations. The variation between the two homozygous genotypic groups was significant for two or all the three traits in each population (*P* < 0.05, 0.01, or 0.0001), indicating a general existence of genotypic effects due to QTLs located in the target region. In the meantime, the variation among different lines of the same genotype was not significant in Z4 but significant for two traits in the three other populations (*P* < 0.05, 0.01, or 0.001), which should be taken into account in testing the genotypic effect. No significant variation was detected for GE interactions, indicating that genotypic variation in the target region had consistent effects on TGW, GL and GW in the two trials which were conducted under distinct ecological conditions.

**Table 1:** Significance levels of the *F*-test’s *p*-values for 1000-grain weight (TGW), grain length (GL) and grain width (GW) in four NIL populations

Source of variation	Z1			Z2			Z3			Z4		
	TGW	GL	GW	TGW	GL	GW	TGW	GL	GW	TGW	GL	GW
Environment (E)	^d^	^d^	^d^	^d^	^d^	^d^	^d^	^d^	^d^	^d^	^d^	^d^
Genotype (G)	^d^	^a^	^a^	ns	^b^	^b^	ns	^a^	^b^	^b^	^d^	ns
Lines within G	ns	^a^	^a^	^a^	ns	^a^	^c^	^b^	ns	ns	ns	ns
G-by-E	ns	ns	ns	ns	ns	ns	ns	ns	ns	ns	ns	ns

^a^, ^b^, ^c^, and ^d^significant at 0.05, 0.01, 0.001 and 0.0001 levels, respectively; *ns* not significant

### Mapping of two QTLs for grain weight in an 835.2-kb region

In each set of the NIL pairs, significant phenotypic difference between the two homozygous genotypic groups is an indication of the presence of a QTL in the given region. Two-way ANOVA was performed to test the genotypic effect, in which the genotypic variation was tested against the variation due to different lines within the same genotypic group. As shown in Table 2, significant genotypic effects were detected in all the four populations, but the traits affected and the allelic directions varied among different populations. These suggested that two or more QTLs for the traits analyzed were located in the target region.

**Table 2:** QTL effects detected in four NIL populations

Population	Segregating region	Trait	Mean trait values				*P*	*A*	*R* ^*2*^ (%)
			Zhejiang trial		Hainan trial				
			NIL^ZS97^	NIL^MY46^	NIL^ZS97^	NIL^MY46^			
Z1	Wn28382 − Wn28447	TGW	25.10	24.87	27.80	27.62	<0.0001	–0.10	14.83
		GL	8.020	7.995	7.972	7.958	0.0135	–0.009	6.02
		GW	3.090	3.083	3.267	3.260	0.0481	–0.004	4.03
Z2	Wn28382 − RM11554	TGW	24.63	24.49	28.02	27.97	0.0869		
		GL	8.040	8.054	7.960	7.992	0.0015	0.011	8.76
		GW	3.070	3.062	3.276	3.262	0.0032	–0.006	8.71
Z3	RM11543 − RM11569	TGW	25.06	25.19	28.10	28.12	0.2174		
		GL	8.040	8.056	7.939	7.960	0.0128	0.010	6.58
		GW	3.080	3.076	3.277	3.267	0.0049	–0.004	6.07
Z4	RM11569	TGW	24.33	24.50	27.91	28.00	0.0025	0.06	7.33
		GL	7.970	8.012	7.855	7.879	<0.0001	0.017	18.27
		GW	3.020	3.026	3.288	3.289	0.6089		

*TGW* 1000-grain weight (g), *GL* grain length (mm), *GW* grain width (mm), *NIL* ^ZS97^, NIL^MY46^ NILs that are Zhenshan 97 and Milyang 46 homozygous in the segregating regions, respectively, *A* additive effect of replacing a ZS97 allele by a MY46 allele, *R* ^*2*^ proportion of phenotypic variance explained by the QTL effect

**Fig. 4:**
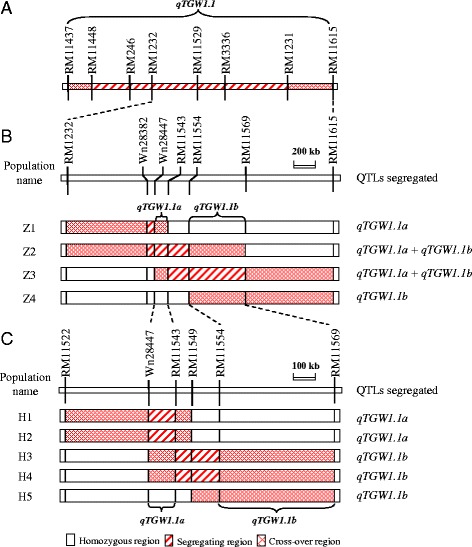
QTLs for grain weight and grain size that were found to be segregated in the rice populations. **a** The *qTGW1.1* region identified previously [17]. **b** Four sets of NIL pairs. **c** Five NIL-F_2_ populations

Three types of genotypes were included in the target region (Fig. 4), *i.e.*, the homozygous region flanked by two homozygous markers, the segregating region flanked by two segregating markers, and the cross-over region flanked by a segregating marker and a homozygous marker. In Z4 that segregated for a single marker RM11569 only (Table 2), no segregating region was shown in the figure. Such a segregating region is in fact the minimum segregating region. The maximum segregating region should include the two flanking cross-over regions, because the areas extending to the two cross-over breakpoints are also segregating regions. Thus a segregating region and its flanking cross-over regions were all included as the segregating region for determining the QTL locations.

For TGW, significant differences between the two genotypic groups were found in Z1 and Z4 but not in the two other populations (Table 2). Since segregating regions of Z1 and Z4 were totally separated from each other (Fig. 4b), two QTLs, *qTGW1.1a* and *qTGW1.1b*, were named following the nomenclature of *qTGW1* in previous studies [17, 19]. The MY46 allele at *qTGW1.1a* and *qTGW1.1b* decreased and increased TGW by 0.10 and 0.06 g, respectively, thus the significant effects may vanish when the two QTLs co-segregated in a population. As shown in Fig. 4b, the segregating regions of Z2 covered the entire segregating region of Z1 and overlapped with the segregating region of Z4, and the segregating regions of Z3 covered the entire segregating region of Z4 and overlapped with the segregating region of Z1. Together with the detection of insignificant genotypic effect in Z2 and Z3, it is indicated that *qTGW1.1a* and *qTGW1.1b* were both segregated in the two populations. Therefore, *qTGW1.1a* was located within the common segregating region of Z1, Z2 and Z3, and *qTGW1.1b* within the common segregating region of Z2, Z3 and Z4. The two regions were the Wn28447 − RM11543 interval for *qTGW1.1a* and the RM11554 − RM11569 interval for *qTGW1.1b* (Fig. 4b).

The effects of *qTGW1.1a* and *qTGW1.1b* were well matched by the effects detected for GL and GW in the four populations (Table 2). In Z1, significant effects were detected for all the three traits with the same allelic direction. The *R*^*2*^ value of 14.83 % for TGW was much higher than the values of 6.02 and 4.03 % for GL and GW, respectively, which was a result of the multiplying effect of GL and GW in grain weight. In Z2 and Z3, significant effects were detected for GL and GW with opposite allelic directions and similar *R*^*2*^, resulting in insignificant effect for TGW. In Z4, significant effects were detected for TGW and GL with the same allelic direction. The *R*^*2*^ was lower for TGW than for GL, presumably due to the opposite effect on GW despite its insignificance.

Due to the small additive effects of *qTGW1.1a* and *qTGW1.1b*, phenotypic distributions of the ZS97 and MY46 lines in each NIL set were not highly distinguishable (Additional files 2, 3 and 4). The most obvious difference between the two genotypes was observed for GL in population Z4 (Additional file 3), followed by TGW in population Z1 (Additional file 2). These results were in accordance with the highest *R*^*2*^ for GL detected in the Z4 population and the second highest *R*^*2*^ for TGW detected in the Z1 population (Table 2).

In conclusion, two QTLs for grain weight, length and width in rice were separated within the interval Wn28447 − RM11569 on the long arm of rice chromosome 1 using the four NIL populations. The *qTGW1.1a* controlled all the three traits with the same allelic direction, thus it had a stronger influence on TGW than on GL and GW. The *qTGW1.1b* mainly affected GL and had a subsequent effect on TGW, having a weaker influence on TGW than on GL. According to the physical positions in the Nipponbare genome, the entire region Wn28447 − RM11569 was 835.2-kb in length, and the distances of the intervals Wn28447 − RM11543 for *qTGW1.1a* and RM11554 − RM11569 for *qTGW1.1b* were 120.4 and 521.8 kb, respectively.

### Validation of *qTGW1.1a* and *qTGW1.1b*

Following the detection of *qTGW1.1a* and *qTGW1.1b* in the four NIL populations, five NIL-F_2_ populations that were derived from five BC_2_F_13_ plants of ZS97^3^/MY46 were used for the QTL validation. Genotypic compositions of these populations in the target region are shown in Fig. 4c. The Wn28447 − RM11543 region for *qTGW1.1a* was a segregating region in H1 and H2, a cross-over region in H3 and H4, and a homozygous region in H5. The RM11554 − RM11569 region for *qTGW1.1b* was a homozygous region in H1 and H2, and a cross-over region in H3, H4 and H5. Thus the H1 and H2 populations were segregated for *qTGW1.1a* only, H5 was not segregated for *qTGW1.1a* but may segregated for *qTGWT1.1b*, and H3 and H4 may segregated for either or both QTLs. Results of the QTL detection using the five NIL-F_2_ populations are presented in Table 3.

**Table 3:** QTL effects detected in five NIL-F_2_ populations

Population	No. of plants	Trait	*LOD*	*A*	*D*	*R* ^*2*^	QTLs matched
H1	237	TGW	3.53	–0.14	0.08	6.89	*qTGW1.1a*
		GL	1.08				
		GW	0.41				
H2	227	TGW	2.09	–0.19	0.04	4.15	*qTGW1.1a*
		GL	0.33				
		GW	0.24				
H3	276	TGW	0.35				
		GL	3.11	0.021	0.00	5.09	*qTGW1.1b*
		GW	0.52				
H4	380	TGW	2.63	0.11	0.09	3.19	*qTGW1.1b*
		GL	3.41	0.020	0.01	4.04	*qTGW1.1b*
		GW	0.22				
H5	279	TGW	3.71	0.12	0.10	5.91	*qTGW1.1b*
		GL	3.37	0.019	0.02	5.38	*qTGW1.1b*
		GW	0.05				

*TGW* 1000-grain weight (g), *GL* grain length (mm), *GW* grain width (mm), *A* additive effect of replacing a ZS97 allele by a MY46 allele, *D* dominance effect, *R* ^*2*^ proportion of phenotypic variance explained by the QTL effect

In H1 and H2, significant QTL effects were detected for TGW but not for the two other traits, confirming the genetic identity of *qTGW1.1a* as a QTL having a stronger effect on TGW than on GL and GW. In the two populations, the MY46 allele acted to decrease grain weight, which was also in accordance with the allelic direction of *qTGW1.1a* detected in the previous experiment. In the trials described in the previous section, the trait values were estimated using replicated trials and *qTGW1.1a* was shown to affect all the three traits, but the effects were much larger for TGW than for GL and GW. Since the trait values were measured based on single plant in the H1 and H2 populations, insignificant effect of *qTGW1.1a* on GL and GW in the two populations could be ascribed to the small QTL effect and large phenotyping error.

In H3, H4 and H5, the effects were significant for GL in all the three populations and significant for TGW in two of the populations, whereas no significant effect was found for GW. In the five cases when a significant effect was detected, the enhancing allele was always derived from MY46. All these were in accordance with the genetic identity of *qTGW1.1b* as a QTL mainly affecting GL. In the meantime, the results also indicate that only *qTGW1.1b* was segregated in the three populations. It was also noted that the additive effects of 0.11 and 0.12 g for *qTGW1.1b* measured in these populations were lower than the values of 0.14 and 0.19 g for *qTGW1.1a* estimated in H1 and H2, which was also in agreement with the relative magnitude detected in the previous experiment.

## Discussion

Since the beginning of this century, great progresses have been achieved in the cloning of major QTLs for yield-related traits in rice, offering the potential for developing a novel breeding strategy using genotypic information [1, 27]. Nevertheless, QTLs that have been cloned and fine-mapped only account for a small proportion of those detected in primary populations [28], and the primary-mapped QTLs themselves have long-been believed to be far less than those truly underlying the trait variation because most experiments have a low power of detecting QTLs with small effects [29, 30]. We have made a continuous effort on the dissection of minor QTLs for grain weight and grain size on the long arm of chromosome 1 in rice [17–19]. In the present study, two QTLs linked in repulsion were separated in an 835.2-kb region. As compared to the ZS97 alleles of *qTGW1.1a* and *qTGW1.1b*, the MY46 alleles had consistent effects in different environments for decreasing and increasing grain weight, respectively. In a few other rice populations, QTLs for grain weight and grain size have been detected in regions in which *qTGW1.1a* and *qTGW1.1b* were contained [16, 31–34]. Since none of these QTLs have been fine-mapped, it remains unknown whether these QTLs are allelic to either *qTGW1.1a* or *qTGW1.1b*.

Separation of closely-linked QTLs has been a great challenge in QTL mapping [35]. When multiple QTLs closely linked are segregated simultaneously in a population, a large population size is required to create sufficient recombination sites and considerable large number of recombinants. Our studies offer a new approach which only requires the detection of few recombinants based on DNA markers located in the region of interest. In the beginning, three BC_2_F_3_ plants having different breakpoints in the 12-Mb target region were identified. Using segregating populations derived from their selfing progenies, two QTLs were identified and named *qTGW1.1* and *qTGW1.2* [17]. Afterwards, six BC_2_F_9_ plants having different breakpoints in the putative *qTGW1.2* region were selected and selfed to produce segregating populations. As significant genotypic effects on TGW were detected in three populations that were segregated in different regions, three QTLs were separated [18]. They were located within the 933.6-kb, 418.8-kb and 2.1-Mb intervals RM11730 − RM11762, RM11781 − RM11800 and RM11800 − RM11885, respectively. Similarly, two QTLs were separated in the present study, located in the 120.4-kb and 521.8-kb intervals Wn28447 − RM11543 and RM11554 − RM11569, respectively. Altogether, five minor QTLs for TGW have been separated in the target region. These results provide new evidence for the returning recognition of the classical concept that a quantitative trait is mainly controlled by many genes with small effects [30].

GE interaction is an important issue in QTL analysis. Due to the bias towards detecting QTLs with large effects, QTLs that have been subjected to careful evaluation for GE interaction are generally those having considerable large effects [36–38]. In our study, the four NIL populations were all planted in two different ecological regions, but the two minor QTLs showed no significant GE interactions. Consistent performance of the two QTLs was also confirmed in the validation experiment conducted using independent samples. These results indicate that minor QTLs for complex traits could stably perform in diverse environments, offering the potential of pyramiding beneficial alleles of multiple minor QTLs through marker-assisted selection. It is also assumable that the insufficient detection of minor QTLs in other studies is largely due to the masking of QTLs having larger effects rather than due to GE interactions.

For *qTGW1.1a* that has been delimitated within a 120.4-kb interval, the QTL region could be soon narrowed down into a region involving a few annotated genes. In view of the small additive effect detected, the difference between the two parental alleles is unlikely to be a result of loss of function or gain of function. Such a small effect may be masked in the presence of other effects such as somaclonal variation produced by plant tissue culture, thus performing complementation test for gene function confirmation might not be applicable. Among other methods for gene determination suggested by the Member of Complex Trait Consortium [39], mutational analysis might be a promising approach. Mutant libraries have been well established in rice [40], which have greatly facilitated the functional genome research in rice. Increasing number of genes isolated from these resources was shown to be the same loci as cloned QTLs [1, 4]. Therefore, rice lines carrying mutant genes in the target QTL regions could be used for the confirmation of the causal QTLs. Alternatively, the quickly evolving gene editing technology using CRISPR/Cas9 [41–43] might provide a great potential to create targeted gene knock-out mutation lines which could be used in the verification of candidate QTLs. If the knock-out results in a large change on grain weight and grain size which could be recovered by the gene isolated from either ZS97 or MY46, then the function could be confirmed.

## Conclusions

Two minor QTLs for grain weight and grain size in rice were separated in an 835.2-kb region on the long arm of chromosome 1, of which *qTGW1.1a* was located within a 120.4-kb interval and *qTGW1.1b* was situated within a 521.8-kb region. They were found to have consistent effects in diverse environments, offering the potential of pyramiding beneficial alleles of multiple minor QTLs through marker-assisted selection and laying a foundation for cloning the minor QTLs. Our results also provide new evidences in support of the classical concept that a quantitative trait is mainly controlled by many genes with small effects.

## Abbreviations

ANOVA, analysis of variance; GE, genotype-by-environment; GL, grain length; GW, grain weight; MY46, milyang 46; NIL, near isogenic line; QTL, quantitative trait locus; SSR, simple sequence repeat; TGW, 1000-grain weight; ZS97, Zhenshan 97

## Additional files

Additional file 1: Table S1. Partition of the phenotypic variance in the four NIL populations. (DOC 38 kb) Additional file 2: Figure S1. Distributions of 1000-grain weight in the four NIL populations grown in Zhejiang and Hainan, respectively. (PPT 99 kb) Additional file 3: Figure S2. Distributions of grain length in the four NIL populations grown in Zhejiang and Hainan, respectively. (PPT 96 kb) Additional file 4: Figure S3. Distributions of grain width in the four NIL populations grown in Zhejiang and Hainan, respectively. (PPT 109 kb)
