# Supplementary material for: Dissection of the qTGW1.1 region into two tightly-linked minor QTLs having stable effects for grain weight in rice
Source: BMC Genet. 2016 Jun 30;17:98. doi: 10.1186/s12863-016-0410-5 (PMC4929766; doi:10.1186/s12863-016-0410-5)
Supplement: Additional file 1: Table S1. — Partition of the phenotypic variance in the four NIL populations. (DOC 38 kb) [file 12863_2016_410_MOESM1_ESM.doc]

**Additional File 1: Table S1. Partition of the phenotypic variance in the four NIL popula**tions

| **NIL** | **Source of variation** | ***df*** | **1000-grain weight** | | |  | **grain length** | | |  | **grain width** | | |
| --- | --- | --- | --- | --- | --- | --- | --- | --- | --- | --- | --- | --- | --- |
|  |  |  | ***SS*** | ***MS*** | ***P*** |  | ***SS*** | ***MS*** | ***P*** |  | ***SS*** | ***MS*** | ***P*** |

Z1 Environment (E) 1 237.4 237.4 <0.0001 0.053 0.053 <0.0001 0.994 0.994 <0.0001

Genotype (G) 1 1.26 1.26 <0.0001 0.011 0.011 0.0135 0.002 0.002 0.0481

Line within G 62 3.65 0.06 0.4804 0.104 0.002 0.0345 0.025 0.000 0.0118

G-by-E 1 0.02 0.02 0.5549 0.001 0.001 0.4622 0.000 0.000 0.7785

Error 62 3.61 0.06 0.065 0.001 0.014 0.000

Z2 Environment (E) 1 378.6 378.6 <0.0001 0.169 0.169 <0.0001 1.314 1.314 <0.0001

Genotype (G) 1 0.31 0.31 0.0869 0.015 0.015 0.0015 0.004 0.004 0.0032

Line within G 62 6.35 0.10 0.0473 0.081 0.001 0.2809 0.028 0.000 0.0198

G-by-E 1 0.05 0.05 0.3884 0.004 0.004 0.0740 0.000 0.000 0.4349

Error 62 4.14 0.07 0.070 0.004 0.017 0.000

Z3 Environment (E) 1 284.5 284.5 <0.0001 0.304 0.304 <0.0001 1.189 1.189 <0.0001

Genotype (G) 1 0.17 0.17 0.2174 0.012 0.012 0.0128 0.002 0.002 0.0049

Line within G 62 6.92 0.11 0.0005 0.111 0.002 0.0038 0.015 0.000 0.6724

G-by-E 1 0.09 0.09 0.1786 0.000 0.000 0.8168 0.000 0.000 0.4764

Error 62 2.97 0.05 0.056 0.001 0.017 0.000

Z4 Environment (E) 1 401.7 401.7 <0.0001 0.480 0.480 <0.0001 2.213 2.213 <0.0001

Genotype (G) 1 0.52 0.52 0.0025 0.039 0.039 <0.0001 0.000 0.000 0.6089

Line within G 62 3.26 0.05 0.5277 0.093 0.002 0.2639 0.017 0.000 0.1394

G-by-E 1 0.04 0.04 0.3705 0.003 0.003 0.1148 0.000 0.000 0.8092

Error 62 3.32 0.05 0.079 0.001 0.013 0.000
