# Supplementary material for: Dissection of the qTGW1.1 region into two tightly-linked minor QTLs having stable effects for grain weight in rice
Source: BMC Genet. 2016 Jun 30;17:98. doi: 10.1186/s12863-016-0410-5 (PMC4929766; doi:10.1186/s12863-016-0410-5)
Supplement: Additional file 2: Figure S1. — Distributions of 1000-grain weight in the four NIL populations grown in Zhejiang and Hainan, respectively. (PPT 99 kb) [file 12863_2016_410_MOESM2_ESM.ppt]

## Slide 1
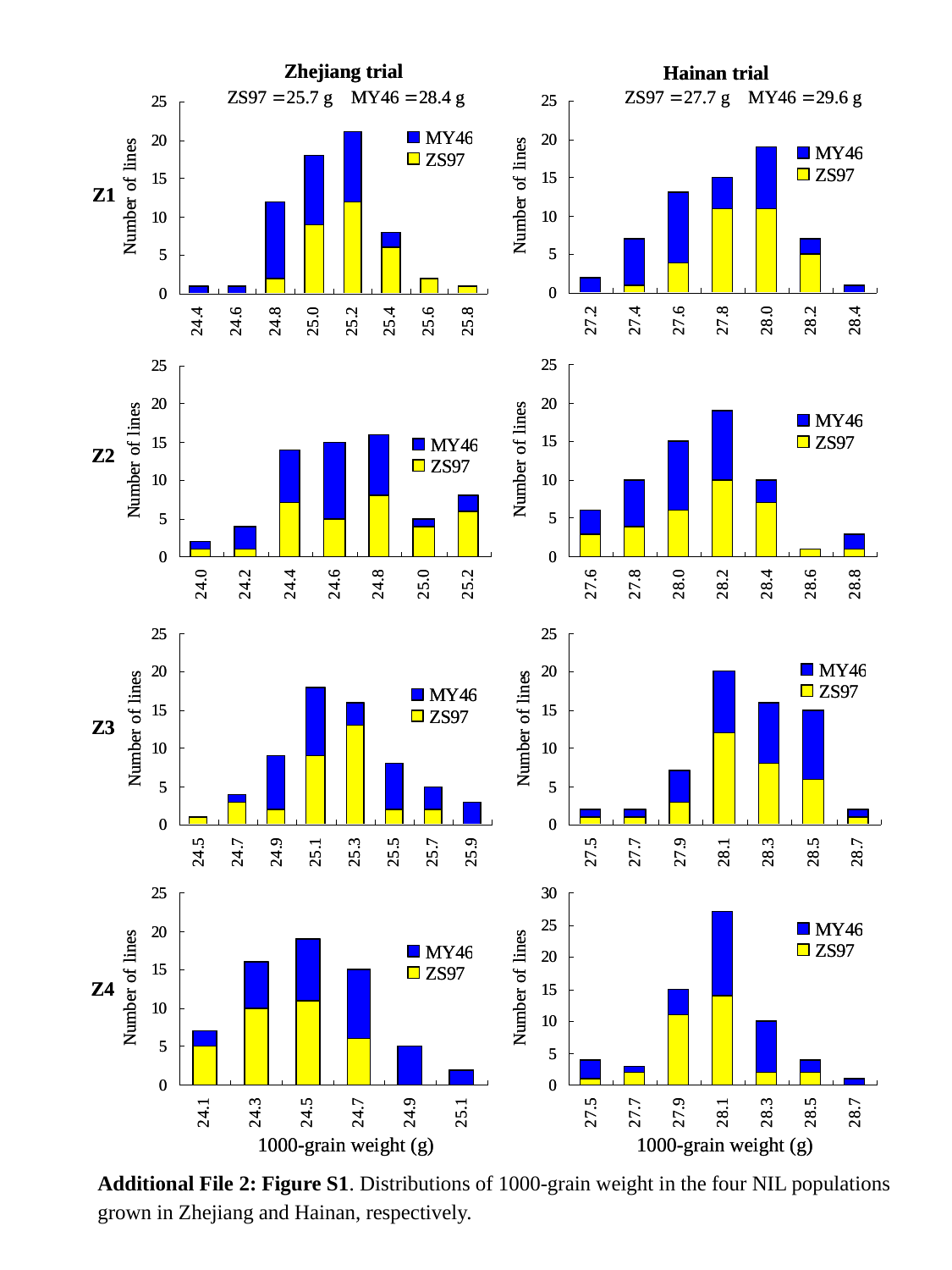

Additional File 2: Figure S1. Distributions of 1000-grain weight in the four NIL populations grown in Zhejiang and Hainan, respectively.
