# Supplementary material for: Dissection of the qTGW1.1 region into two tightly-linked minor QTLs having stable effects for grain weight in rice
Source: BMC Genet. 2016 Jun 30;17:98. doi: 10.1186/s12863-016-0410-5 (PMC4929766; doi:10.1186/s12863-016-0410-5)
Supplement: Additional file 3: Figure S2. — Distributions of grain length in the four NIL populations grown in Zhejiang and Hainan, respectively. (PPT 96 kb) [file 12863_2016_410_MOESM3_ESM.ppt]

## Slide 1
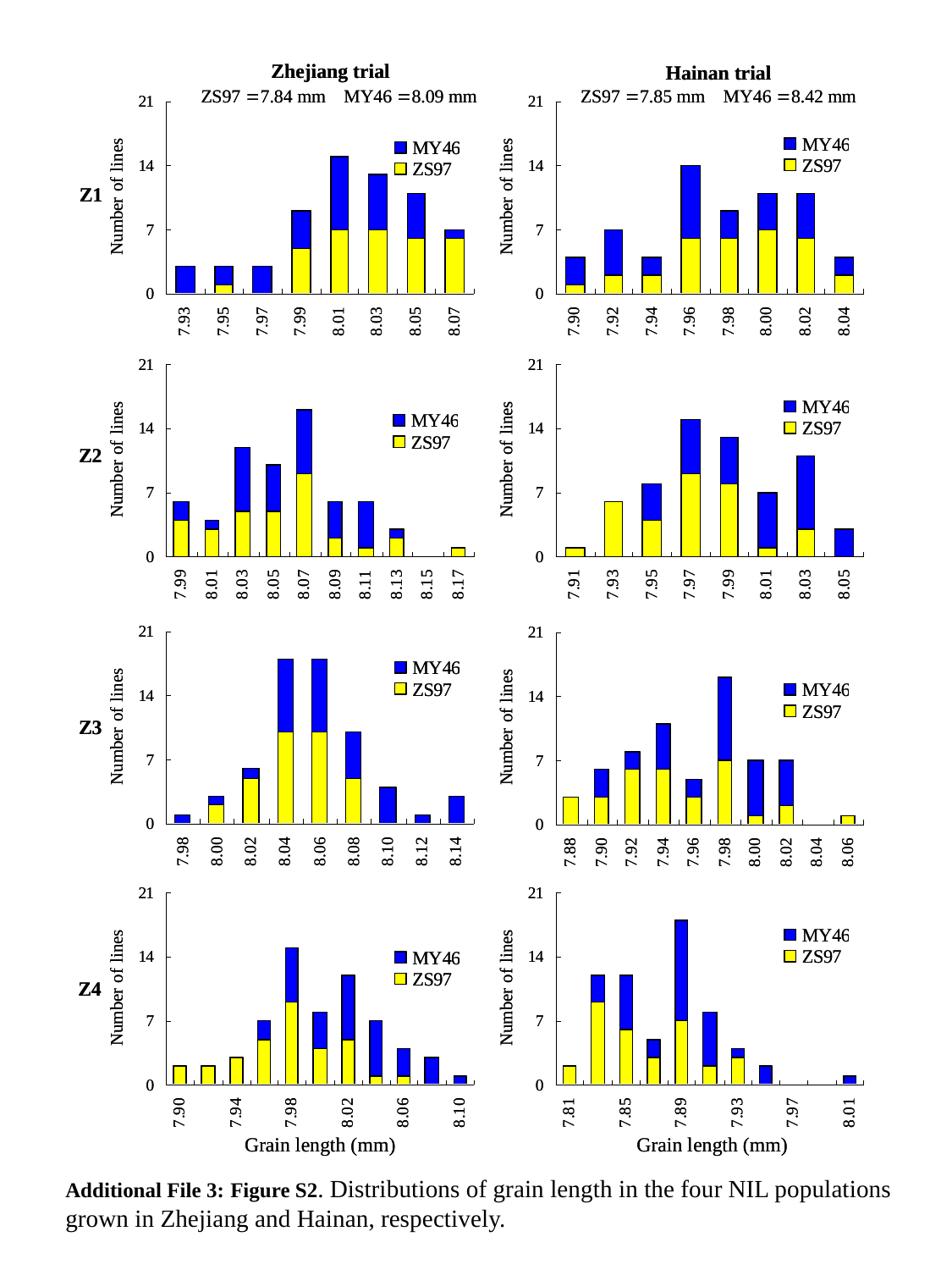

Additional File 3: Figure S2. Distributions of grain length in the four NIL populations grown in Zhejiang and Hainan, respectively.
